# Developing cardiac digital twin populations powered by machine learning provides electrophysiological insights in conduction and repolarization

**DOI:** 10.1038/s44161-025-00650-0

**Published:** 2025-05-16

**Authors:** Shuang Qian, Devran Ugurlu, Elliot Fairweather, Laura Dal Toso, Yu Deng, Marina Strocchi, Ludovica Cicci, Richard E. Jones, Hassan Zaidi, Sanjay Prasad, Brian P. Halliday, Daniel Hammersley, Xingchi Liu, Gernot Plank, Edward Vigmond, Reza Razavi, Alistair Young, Pablo Lamata, Martin Bishop, Steven Niederer

**Affiliations:** 1 National Heart and Lung Institute, Imperial College London, London, UK; 2 Department of Biomedical Engineering, School of Imaging Sciences and Biomedical Engineering, Kingʼs College London, London, UK; 3 Alan Turing Institute, British Library, London, UK; 4 Institute for Biomedical Engineering, ETH Zurich and University Zurich, Zurich, Switzerland; 5 Cardiovascular Magnetic Resonance Unit, Royal Brompton and Harefield Hospitals, Guy’s and St. Thomas’ National Health Service Foundation Trust, London, UK; 6 Anglia Ruskin School of Medicine & MTRC, Anglia Ruskin University, Chelmsford, UK; 7 King’s College Hospital NHS Foundation Trust, London, UK; 8 Scientific Computing Department, Science and Technology Facilities Council, Harwell, UK; 9 Gottfried Schatz Research Centre-Biophysics, Medical University of Graz, Graz, Austria; 10 BioTechMed-Graz, Graz, Austria; 11 University of Bordeaux, CNRS, Bordeaux, France; 12 IHU Liryc, Fondation Bordeaux Université, Talence, France

**Keywords:** Computational models, Predictive markers, Biomedical engineering, Machine learning

## Abstract

Large-cohort imaging and diagnostic studies often assess cardiac function but overlook underlying biological mechanisms. Cardiac digital twins (CDTs) are personalized physics-constrained and physiology-constrained in silico representations, uncovering multi-scale insights tied to these mechanisms. In this study, we constructed 3,461 CDTs from the UK Biobank and another 359 from an ischemic heart disease (IHD) cohort, using cardiac magnetic resonance images and electrocardiograms. We show here that sex-specific differences in QRS duration were fully explained by myocardial anatomy while their myocardial conduction velocity (CV) remains similar across sexes but changes with age and obesity, indicating myocardial tissue remodeling. Longer QTc intervals in obese females were attributed to larger delayed rectifier potassium conductance ${G}_{\rm{KrKs}}$. These findings were validated in the IHD cohort. Moreover, CV and ${G}_{\rm{KrKs}}$ were associated with cardiac function, lifestyle and mental health phenotypes, and CV was also linked with adverse clinical outcomes. Our study demonstrates how CDT development at scale reveals biological insights across populations.

## Abstract (web summary)

Qian et al. used cardiac magnetic resonance images and electrocardiograms from the UK Biobank and an ischemic heart disease cohort and developed more than 3,500 cardiac digital twins. They demonstrate that the myocardial anatomy causes differences in the QRS duration among sexes while age and body weight alter potassium conductance and myocardial conduction velocity.

## Main

Large-cohort multi-modality cardiovascular imaging and diagnostic datasets are increasingly available and are being used to link heart anatomy and function with physiological, lifestyle and clinical outcomes. Although providing hypothesis-generating correlations, they have been less successful at identifying the underlying mechanisms that drive these correlations. This is, in part, because they are restricted to the analysis of observed attributes and do not identify the underlying physiology that may explain the observed correlations.

A strategy to alleviate this limitation is the personalization of cardiac digital twins (CDTs)^1^. CDTs provide physics-constrained and physiology-constrained in silico representations of specific individuals. They are personalized by integrating multimodal data, enabling multi-scale structure and function to be inferred from clinical measurements. The model parameters that explain the data become the attributes that describe the underlying physiology. Early forms of CDTs have shown great potential in supporting clinical decision-making and providing tailored therapies, as in prospective studies of ventricular tachycardia^2^, atrial fibrillation^3^ and cardiomyopathy^4^.

However, creation of CDTs is associated with challenges. Complex data processing and specialist methodology and requirement of large computational resources are required, which limit their broad adoption in both industrial and clinical settings. Presently, studies are constrained to working with small patient cohorts with $\le 100$ patients^5^, limiting their application and scalability to large population datasets.

The creation of CDTs involves two crucial steps. First is the construction of the anatomical twin—the computational replica of the anatomical structures of each individuals’s heart from medical images. Previously, semi-automatic workflows of heart mesh generation were developed^6^, demanding substantial computational resources and considerable manual interventions by trained experts. The second is to build the functional twin—that is, identifying bespoke electrophysiological (EP) parameters that replicate clinical measurements, for example electrocardiograms (ECGs). This step is often more challenging, requiring numerous computationally intensive simulations to calibrate the multi-scale parameters, based on the specific biophysical fidelity needed. Parameter personalization remains an ongoing challenge, with most modeling studies relying on ‘average’ parameters derived from the literature^7,8^.

The feasibility of generating CDTs at scale hinges on the development of a computationally and time-efficient automated workflow for both CDT creation steps. Recent advances in image segmentation, including nnU-Net^9^ and Atlas-based approaches^10^, provide robust and precise three-dimensional anatomical structures within abbreviated timeframes, facilitating the rapid creation of anatomical meshes from medical images. The emergence of surrogate models as novel statistics and machine learning tools—for example, Gaussian process emulators (GPEs)^11^—provides a low-cost statistical representation of computationally expensive models. Such surrogate models enable global sensitivity analysis (GSA) to identify important model parameters and, therefore, constrain the viable parameter space, which can reduce the number of simulations necessary for model calibration.

In this study, we developed a methodology that integrates multi-modality data from the UK Biobank (UKBB)^12^ within a CDT framework and demonstrates the feasibility of CDT creation at scale, to our knowledge for the first time. Within this framework, we inferred myocardial conduction velocities (CVs) and an integrated measure of rapid and slow delayed rectifier potassium conductance ${G}_{\rm{KrKs}}$ for each CDT from their QRS duration (QRSd) and corrected QT (QTc) interval. We then reported how CV and ${G}_{\rm{KrKs}}$ vary across sex, body mass index (BMI) and age, together with imaging and ECG-derived phenotypes (biventricular myocardial mass, QRSd and QTc, respectively). We conducted a phenome-wide association study (PheWAS) to explore their relationships with other phenotypes reported in the UKBB, followed by assessing their ability to independently predict clinical outcomes.

## Results

### Anatomical and functional CDT generation workflow

**Fig. 1:**
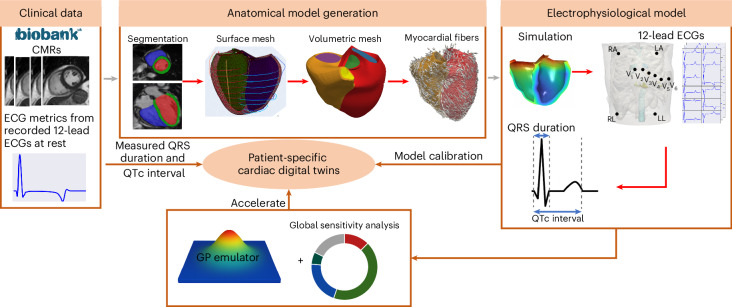
The automated anatomical and functional (EP) CDT generation workflow. The anatomical models are personalized finite element meshes with physiological-detailed myocardial fibers constructed from the short-axis and long-axis heart images in the UKBB following the steps of segmentations, surface meshes and volumetric meshes construction as well as myocardial fiber generation. The functional CDT workflow is to replicate the EP activities within the anatomical models to match the QRSd and QTc interval from the clinically measured 12-lead ECGs. Reproduced by kind permission of the UKBB.

The CDTs were built from the UKBB magnetic resonance imaging (MRI) and ECG datasets as depicted in Fig. 1. Each CDT simulates the ECG, as measured by the QRSd and QTc interval. The CDT consists of a model of the heart anatomy, the preferred myofiber architecture, a fast endocardial conducting layer, the location of the activating Purkinje fascicles, the activation timing, the tissue conductivity, the degree of anisotropy, spatial heterogeneity of ionic conductance and the location of the virtual ECG electrodes. The personalization involved the generation of the bespoke biventricular anatomy from MRI and the inference of the CV and ${G}_{\rm{KrKs}}$ that replicates the QRSd and QTc interval, respectively.

**Fig. 2:**
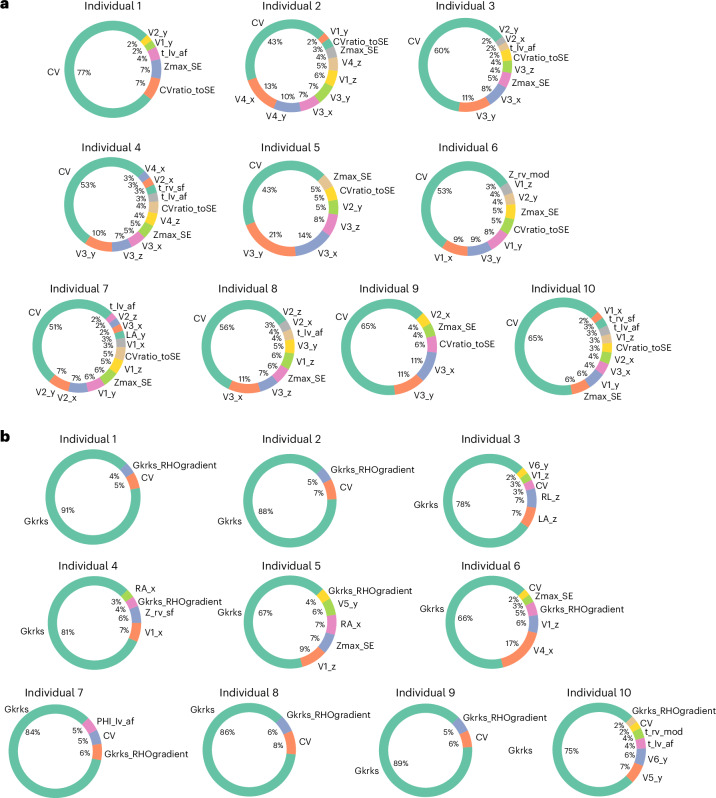
GSA results. The total effects of all parameters explain the variance of interested outputs in 10 individuals sampled from the cohort based on sex, age and BMI. **a**, GSA results for QRSd. **b**, GSA results for QTc interval. Gkrks_RHOgradient, the transmural gradients of ${{{G}}}_{{\rm{KrKs}}}$. Gkrks_conductance, the scaling factor of baseline ${{{G}}}_{{\rm{KrKs}}}$. Note that only parameters with more than 2% impact on the output are included in the plots. Source data

**Extended Data Fig. 1:**
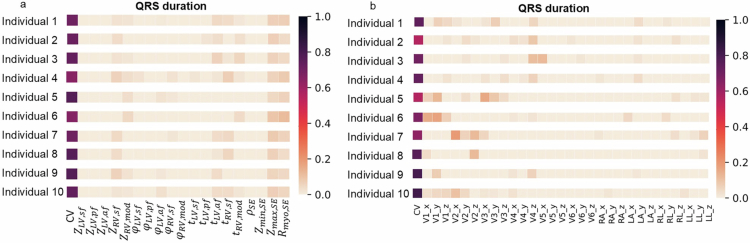
Global sensitivity analysis results for the electrophysiological and ECG electrode positions parameters on QRS duration. The total effects of input EP parameters explain the variance of output QRS duration in 10 representative individuals sampled from the cohort based on sex, age and BMI. **a** is for only tissue-level EP parameters and **b** is for the 30 ECG electrodes’ location parameters combining with the most important tissue-level parameter from **a**. Source data

**Extended Data Fig. 2:**
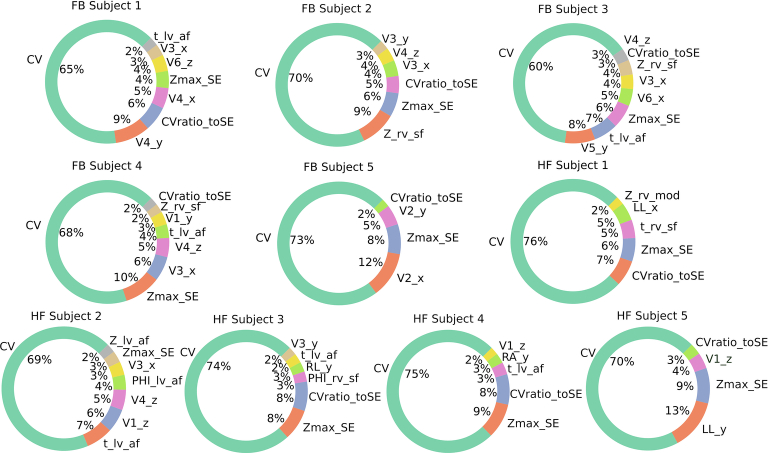
Global sensitivity analysis results for the whole parameter set on QRS duration in 10 representative individuals with pathology. The total effects of all input parameters on QRS duration were assessed. Note that only parameters with >2% impact on the output are included in the sensitivity plots. Source data

The functional personalization required a preliminary analysis to determine which parameters could be inferred from the available data and which needed to be set to reference prior values. Thus, we performed two GSAs. In 10 representative individuals sampled across sex, BMI and age, we calculated the sensitivity of the QRSd prediction to 20 tissue-level physiological parameters or 30 ECG electrode positions combined with the most important tissue-level property (Supplementary Tables 1 and 2). Notably, we found that CV has the dominant effect on QRSd, accounting for 71.9% ± 4.5%, across all EP parameters, and $68.9\pm 9 \%$, across ECG electrode positions (Extended Data Fig. 1). The GSA results on the whole parameter set (Fig. 2a) are consistent with the separate analysis above that CV explains most of the variation in QRSd ($56.6\pm 10 \%$). Additionally, we also identified 10 representative individuals with pathology (Supplementary Table 3) and performed GSA on the whole parameter set for those individuals and observed consistent dominant effect of CV on QRSd ($70\pm 4.9 \%$) (Extended Data Fig. 2).

**Extended Data Fig. 3:**
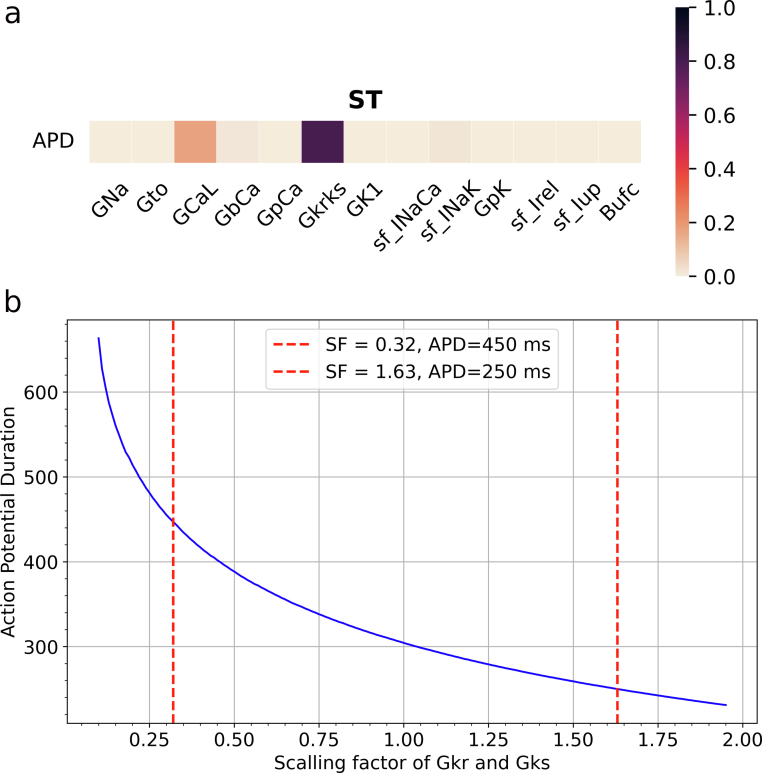
Parameter analysis for repolarization electrophysiological model framework. **a**. Global sensitivity analysis results for the ionic parameters on action potential duration (APD: the key cellular feature to QTc interval). **b**. Finding the ranges of the baseline ${G}_{{KrKs}}$ conductance based on maximum APD ranges measured physiologically. Source data

Second, we investigated the sensitivity of the QTc interval prediction by adding repolarization parameters to the parameter set. In human cardiac myocyte models, the relative contribution of ${I}_{\rm{Kr}}$ and ${I}_{\rm{Ks}}$ to repolarization remains debated^13^. To recognize this ambiguity, we introduce a common scaling parameter, ${G}_{\rm{KrKs}}$, for the slow and rapid delayed rectifier potassium conductances, given that we are not able to differentiate between their individual effects on QTc. First, we performed a cellular GSA for all ionic parameters on action potential duration (APD) (the cellular analog to QTc interval) and found that ${G}_{\rm{KrKs}}$ represents 80% of the sensitivity (Supplementary Table 4 and Extended Data Fig. 3a). We then performed a GSA on QTc interval in an augmented parameter set including three repolarization parameters: the scaling factor of baseline ${G}_{\rm{KrKs}}$ (referred to as ${G}_{\rm{KrKs}}$ for simplicity) and its transmural and apex-to-base gradients. The GSA results reveal that ${G}_{\rm{KrKs}}$ is the most important parameter affecting QTc interval by $80.5\pm 8.4 \%$ (Fig. 2b).

**Extended Data Fig. 4:**
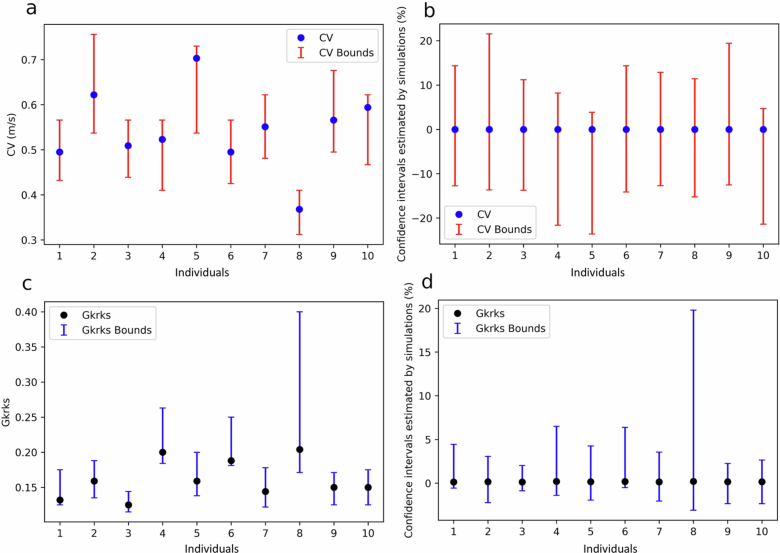
Uncertainty quantification for the 10 representative individuals. **a**, the fitted conduction velocity (CV) values for 10 subjects (blue circles) with uncertainty ranges (red error bars) derived from the 5th and 95th percentiles of simulated QRS duration distribution due to the variability of the other 49 parameters affecting QRS duration. **b**. the corresponding confidence interval of fitted CVs in %, with the median lower and upper bounds across the 10 subjects of −12.1% and +13.9%. **c**, the fitted ${G}_{{KrKs}}$ values for 10 subjects (blue circles) with uncertainty ranges (red error bars) derived from the 5th and 95th percentiles of simulated QTc distribution due to the variability of the other 52 parameters affecting QTc. **d**. the corresponding confidence interval of fitted ${G}_{{KrKs}}$ in %, with the median lower and upper bounds across the 10 subjects of −14.2% and +24.7%. Source data

Given that CV and ${G}_{\rm{KrKs}}$ were identified as the key parameters that explain most of the variation in QRSd and QTc interval, we chose to calibrate the models by applying a computational-efficient bisection method to search for, first, the personalized CV (constrained by physiological measurements in literature^14,15^) and, then, ${G}_{\rm{KrKs}}$ (constrained by values derived to match the range of physiological-measured maximum ventricular APD in literature^16–19^; Extended Data Fig. 3b), to match with their measured QRSd and QTc interval from the 12-lead ECGs at rest. The CDT calibration process assumed that all other parameters were set to reference prior values taken from the literature, introducing an estimated uncertainty of −12.1% to +13.9% around the ‘true’ CV and −14.2% to +24.7% around the ‘true’ ${G}_{\rm{KrKs}}$ (Extended Data Fig. 4).

We used the 4,326 first participants with consent from the UKBB who had adequate geometrical information^10^ along with reported QRSd, QTc, sex, age, BMI/weight and height information. Of these, 3,942 (91.1%) and 3,461 (80%) were successfully processed through the anatomical and functional personalization workflows, respectively. Summary participant characteristics are shown in Supplementary Table 5.

We calculated the left and right ventricular end-diastolic volumes (LVEDV and RVEDV) for our meshes and compared these to values derived from manual segmentations as a reference. The LVEDV and RVEDV calculated from the mesh using numerical integration agreed well with the volume derived from manual segmentations. The absolute and relative differences of LVEDV and RVEDV were $-12.7\pm 10.3\;\rm{ ml}$ (*P* < 0.005) and $-20.8\pm 12.9\;\rm{ml}$ (*P* < 0.005), respectively.

### Model validation

**Extended Data Fig. 5:**
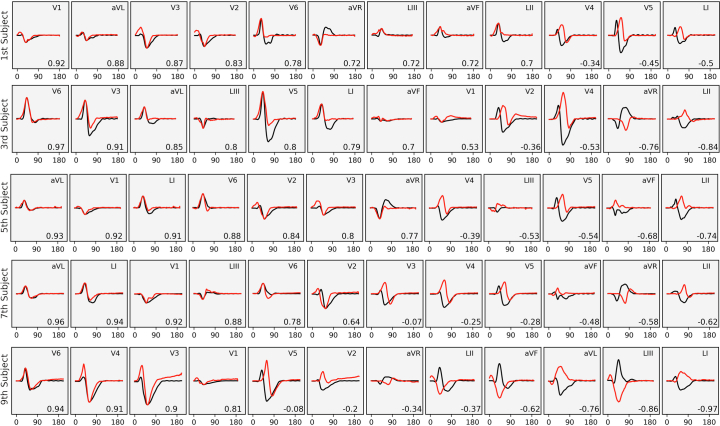
Comparison of simulated 12 lead ECGs (black) with recorded ECGs (red) in the UK biobank of the five example representative individuals. It shows five example cases which are selected as every second case ranked from best to worst matched to recordings (due to the UK biobank restrictions). Each simulated lead ECGs were scaled to have the same maximum absolute amplitude as the corresponding recorded lead ECGs in $\mu V$. All lead simulated and recorded ECGs were temporally aligned by matching the timepoints that the maximum voltage energy were achieved. Then the correlation scores for each pair of simulated and recorded lead ECGs were computed as shown at the bottom right corner of the plots and the 12 lead ECGs were listed in descending order of their correlation scores.

**Extended Data Fig. 6:**
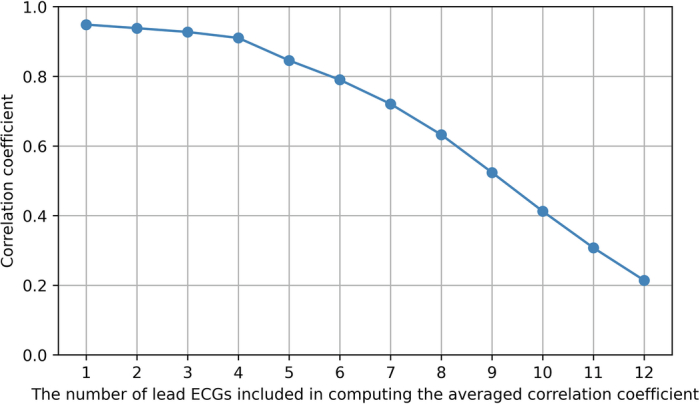
Correlations between simulated and recorded ECGs of the ten representative individuals. The averaged correlation coefficients (r) of simulated and recorded ECGs versus the number of lead ECGs included in computing the averaged $r$. Source data

To validate the CDT workflow, we compared the QRS morphology in simulated ECGs against the measured ECGs in the 10 representative individuals. As the ECGs were simulated from a reference torso and heart location, we do not expect perfect matches in all leads. We adopted a lead-to-lead comparison approach used to compare ECGs clinically^20^. To quantify the ability of the model to replicate the ECG morphology, we plotted the simulated 12-lead ECGs (filtered, scaled and temporally aligned) against measured ECG, and correlation coefficients ($r$) were calculated. Extended Data Fig. 5 shows the comparison for five example cases that are selected as every second case ranked from best to worst matched, with plots for each case ordered in descending order of the correlation coefficients. We found that 56% of ECG leads in all individuals matched with the recordings well ($r > 0.5$). Extended Data Fig. 6 shows the averaged $r$ of the best correlated ECG leads as the number of leads considered increases. The maximum average $r$ considering only the best correlated ECG leads was 0.95, whereas its value dropped to 0.79 when considering the top six ECG leads. We also found the correlation of precordial leads to be higher than limb leads (precordial: $0.395\pm 0.648$ versus $0.033\pm 0.763$, *P* = 0.0003). In contrast to correlations in 12-lead ECGs, the correlations between the magnitude of simulated and recorded dipoles are higher as 0.87$\pm 0.09$, and their deviation $\theta$ in dipole orientations is small as $15.0^\circ \pm 10.7^\circ$ (Supplementary Table 6). Regarding the repolarization, as we fitted QTc values, it was not appropriate to perform a comparison of simulated versus measured QT morphologies.

**Fig. 3:**
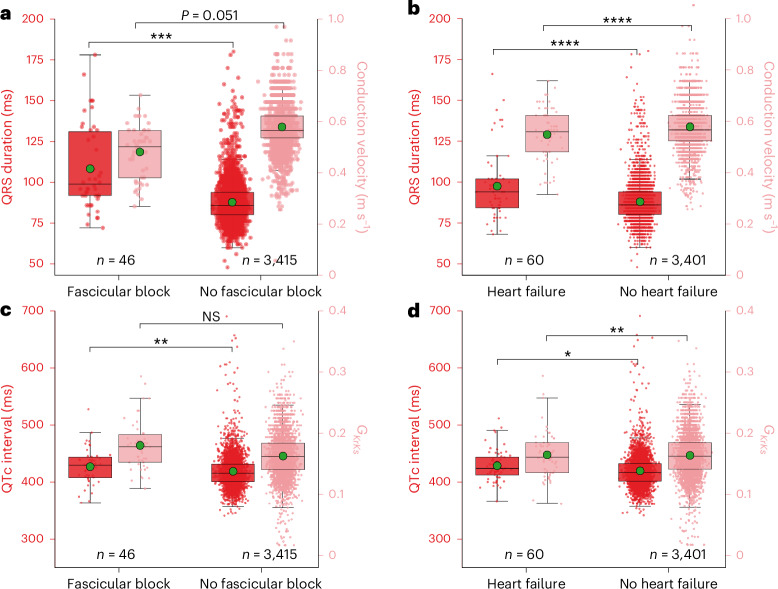
Comparison of ECG and CDT-derived phenotypes of pathological individuals having FB or HF with their control counterparts. Comparison of QRSd and CV (**a** and **b**) and QTc interval and delayed rectifier potassium conductance ‘${{{G}}}_{{\rm{KrKs}}}$’ (**c** and **d**) for groups of participants afflicted with FB or HF and non-afflicted counterparts. The centerline within each box represents the median (50th percentile), and the box bounds correspond to the interquartile range (IQR), with the lower and upper edges indicating the 25th and 75th percentiles, respectively. The whiskers extend to the minimum and maximum values within 1.5 times the IQR from the quartiles. The green dots indicate their means. The corresponding *P* values are from the two-sided Mann–Whitney *U*-test. *P < 0.05; **P < 0.01; ***P < 0.001; ****P < 0.0001. NS, not significant.

To further validate our modeling approach, we investigated whether individuals with pathologically slow conduction or abnormal repolarization—for instance, caused by fascicular block (FB) or heart failure (HF)—can be differentiated through their personalized CVs or baseline ${G}_{\rm{KrKs}}$ conductance. Those individuals were identified using the summary diagnoses for hospital inpatients in the UKBB with the specific disease types as shown in Supplementary Table 7. Figure 3 shows that individuals with FB (*N* = 46) had 16.8% lower CV (0.482 ± 0.11 m s^−1^ versus 0.579 ± 0.08 m s^−1^, $P=0.051$), 23.3% longer QRSd (108.4 ± 26.0 ms versus 87.9 ± 12.6 ms, $P={3\times 10}^{-9}$), 2.4% longer QTc interval (428.5 ± 27.4 ms versus 419.3 ± 32.7 ms, $P=0.003$) and similar baseline ${G}_{\rm{KrKs}}$ conductance (0.164 ± 0.04 versus 0.163 ± 0.04, $P=0.95$) compared to their control counterparts. In individuals with HF (*N* = 60), we observed 5.2% slower CV (0.548 ± 0.10 m s^−1^ versus 0.578 ± 0.08 m s^−1^, $P={1\times 10}^{-4}$), 10.7% longer QRSd (97.6 ± 20.8 ms versus 88.1 ± 12.8 ms, $P={3\times 10}^{-9}$), 2% longer QTc interval (427.7 ± 31.8 ms versus 419.4 ± 32.6 ms, $P=0.02$) and 10% smaller baseline ${G}_{\rm{KrKs}}$ conductance (0.18 ± 0.04 versus 0.163 ± 0.04, $P=0.005$) compared to their control counterparts.

### Comparison for different sex, BMI and age groups

**Fig. 4:**
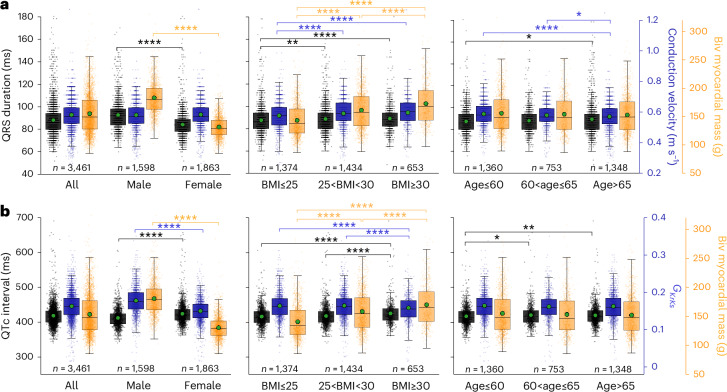
Comparison of imaging, ECG and CDT-derived phenotypes for different sex, age and BMI groups. Box plots of QRSd and CV (**a**) and QTc interval and delayed rectifier potassium conductance ‘${{{G}}}_{{\rm{KrKs}}}$’ (**b**) with myocardial mass for different groups of sex, BMI and age. The centerline within each box represents the median (50th percentile), and the box bounds correspond to the interquartile range (IQR), with the lower and upper edges indicating the 25th and 75th percentiles, respectively. The whiskers extend to the minimum and maximum values within 1.5 times the IQR from the quartiles. The green dots indicate their means. The corresponding *P* values are from the two-sided Mann–Whitney *U*-test. *P < 0.05; **P < 0.01; ***P < 0.001; ****P < 0.0001. Biv, biventricular.

We compared QRSd, CV, QTc interval, baseline ${G}_{\rm{KrKs}}$ conductance and biventricular myocardial mass, categorized into different groups of sex, BMI and age as shown in Fig. 4. The QRSd was 9.4% longer in males (92.9 ± 13.2 ms versus 84.2 ± 11.5 ms, $P={6\times 10}^{-113}$). This is consistent with males tending to have larger hearts (male: 180.7 ± 28.2 g versus 131 ± 18.9 g, $P < {1\times 10}^{-324}$). However, CVs were the same in men and women (0.576 ± 0.09 m s^−1^ versus 0.579 ± 0.08 m s^−1^, *P* = 0.4). We observed 3% longer QTc intervals in females than males (425.1 ± 33.5 ms versus 413 ± 30.3 ms, $P={2\times 10}^{-41}$), reflected in the ion channel level by a drop in ${G}_{\rm{KrKs}}$ conductance by 18% in females (0.151 ± 0.04 versus 0.178 ± 0.04, $P={2\times 10}^{-120}$).

QRSd was longer for overweight and obese ($25\le \rm{BMI}\le 30$ and $\rm{BMI}\ge 30$) compared to healthy ($\rm{BMI}\le 25$) groups (overweight: 88.6 ± 12.9 ms and obese: 89 ± 12.8 ms versus healthy: 87.5 ± 13.2 ms, *P* = 0.005 and *P* = 0.0005). Again, this increase in QRSd with BMI was reflected with a corresponding increase in heart size (healthy: 141.4 ± 29.2 g, overweight: 158.7 ± 33.5 g, obese: 170.2 ± 36.2 g, all $P < {1\times 10}^{-10}$). In contrast with sex, this increase in QRSd was also associated with a progressive increase in CVs (healthy: 0.569 ± 0.08 m s^−1^ versus overweight and obese: 0.582 ± 0.08 m s^−1^ and 0.587 ± 0.09 m s^−1^, $P={2.7\times 10}^{-6}\; \;{\rm{and}}\; \;{3.4\times 10}^{-7}$), which suggests a compensatory mechanism for the increment of heart size (that is, CV is increased as a mechanism to reduce QRSd when the heart needs to grow to meet the larger demand of an increased BMI). Similar to QRSd, QTc intervals were longer in the obese group (obese: 425.8 ± 4 ms versus overweight: 418.7 ± 3.1 ms and healthy: 417.3 ± 3 ms, $P < {2\times 10}^{-6}$), consistent with the observed reduced ${G}_{\rm{KrKs}}$ in the obese group (0.159 ± 0.05 versus 0.165 ± 0.04 versus 0.164 ± 0.04, $P < {7\times 10}^{-5}$).

**Extended Data Fig. 7:**
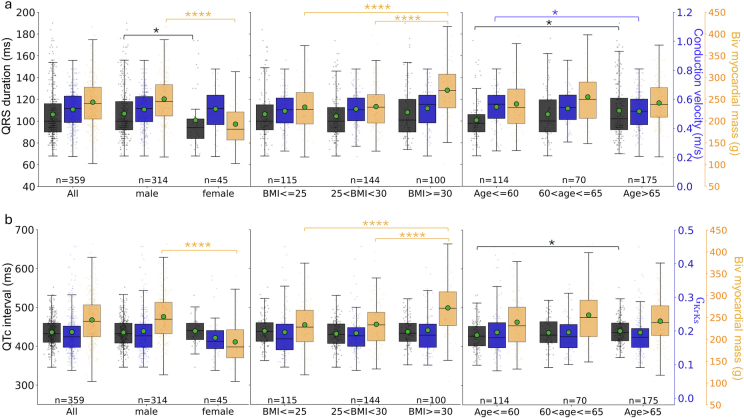
Boxplots for different groups of sex, BMI and age in a clinical cohort of patients with ischemic heart diseases. (**a**) shows QRS duration and conduction velocity; (**b**) shows QTc interval and delayed rectifier potassium conductance ${G}_{{KrKs}}$ with myocardial mass. The centerline within each box represents the median (50th percentile), while the box bounds correspond to the interquartile range (IQR), with the lower and upper edges indicating the 25th and 75th percentiles, respectively. The whiskers extend to the minimum and maximum values within 1.5 times the IQR from the quartiles. The green dots indicate their means. The corresponding P values are from the two-sided Mannwhitney U-test. *P < 0.05; **P < 0.01; ***P < 0.001; ****P < 0.0001. Source data

QRSd increased with aging, which was substantial when comparing the $\rm{Age}\le 60$ group (87.3 ± 11.6 ms) with $\rm{Age}\ge 65$ (89.3 ± 14.7 ms, *P* = 0.02). This QRS increase is consistent with a substantial CV decrease observed between $\rm{Age}\ge 65$ and the other age ranges (0.569 ± 0.09 versus $\rm{Age}\le 60$: 0.586 ± 0.08 versus $60\le \rm{Age}\le 65$: 0.578 ± 0.08 m s^−1^, *P* = ${2\times 10}^{-6}$ and *P* = 0.03), although the myocardial mass stayed unchanged (155.5 ± 35.7 versus 153.8 ± 34.4 versus 152.5 ± 32.6 g). QTc interval was also increased with aging ($\rm{Age}\le 60$: 417.8 ± 0.3 versus $60\le \rm{Age}\le 65$: 420.9 ± 0.4 versus $\rm{Age}\ge 65$: 420.4 ± 0.3 ms, *P* = 0.05 and *P* = 0.001), whereas no significance was observed in their ${G}_{\rm{KrKs}}$. By inferring CV and ${G}_{\rm{KrKs}}$, it is possible to determine when changes in QRSd or QTc interval with age, sex or BMI are caused by changes in cardiac anatomy or biological material properties. Further model validation was conducted by inferring the personalized CV and ${G}_{\rm{KrKs}}$ from QRSd and QTc for a clinical cohort of 359 patients with ischemic heart disease (IHD) (Supplementary Table 8). We observed similar trends as above with several comparisons reaching statistical significance because of the smaller sample size, and the details can be found in Extended Data Fig. 7.

### PheWAS

We performed a PheWAS to explore the correlations between the multimodal and CDT-derived phenotypes and UKBB-reported phenotypes in categories: pulse wave analysis (PWA), LV size and function (automatically derived from heart MRIs), abdominal composition, medication (medical treatment received), primary demographics, early-life information, self-reported medical conditions, lifestyle diet, alcohol, smoking, physical activity, physical measures, education and employment, mental health and clinical outcomes of seven common diseases categorized from summary diagnoses in the UKBB (Supplementary Table 7).

**Fig. 5:**
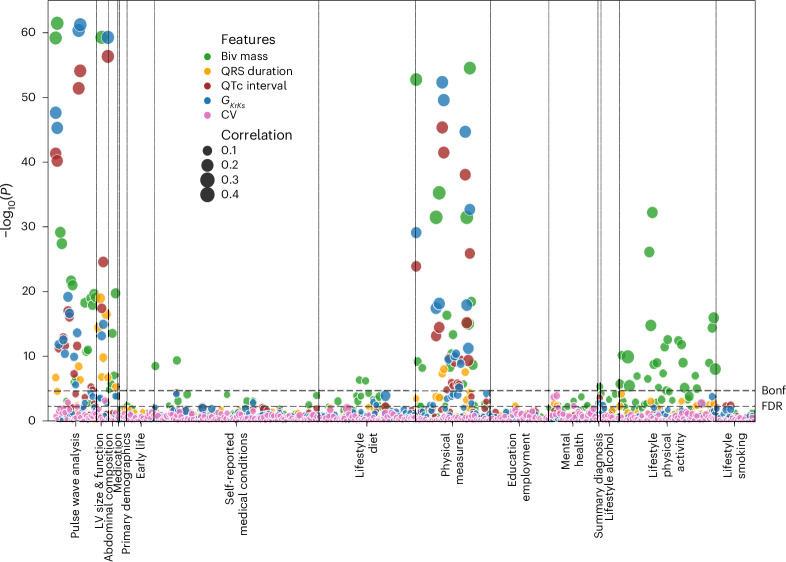
Manhattan plot showing the ${\mathbf{-log}}_{\mathbf{10}}{\boldsymbol{P}}$ (two-sided *t*-test) for correlations among the QRSd, CV, QTc interval, delayed rectifier potassium conductance ${\mathbf{G}}_{\mathbf{KrKs}}$, myocardial mass and UKBB-reported phenotypes. The size of the dots indicates the absolute Spearman correlation coefficient. The dashed horizontal lines are the Bonferroni threshold (Bonf) and the FDR (α = 0.05). Note that the plot is clipped at 65 for better visualization. Biv, biventricular. Source data

**Extended Data Fig. 8:**
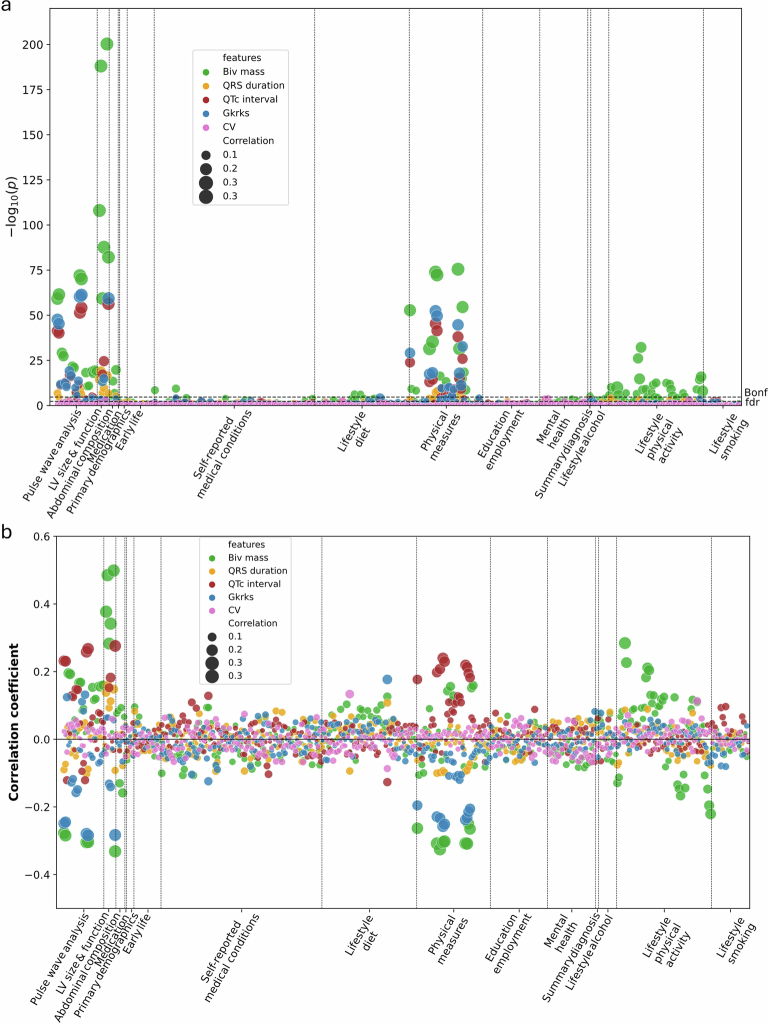
Manhattan plot for correlations between the multimodal phenotypes and phenotypes in the UK biobank. **a**. ${-\log }_{10}P$ (two-sided t-test) for correlations between the multimodal phenotypes and phenotypes in the UK biobank. The size of the dots indicates the absolute Spearman’s correlation coefficient. The dashed horizontal lines are the Bonferroni threshold (Bonf) and the false-discovery rate (fdr) (α = 0.05). **b**. the corresponding correlation coefficients. The size of the dots indicates the absolute Spearman’s correlation coefficient. Source data

Figure 5 shows the Manhattan plot of the univariate Spearman correlation *P* values (two-sided) between *M* = 5 multimodal phenotypes and *N* = 456 UKBB phenotypes for *M* × *N* = 2,280 times, with 152 correlations reaching the Bonferroni threshold for multiple comparisons (${P}_{\rm{bonf}}={2.2\times 10}^{-5}$ for α = 0.05) and 258 correlations reaching the false discovery rate (FDR) threshold (${P}_{\rm{fdr}}=0.005$ for α = 0.05). For the full plot and their correlation coefficients in the Manhattan plots, see Extended Data Fig. 8.

QRSd was considerably associated with many cardiac structural, functional and hemodynamic phenotypes, including LV end-diastolic, end-systolic and stroke volumes, cardiac index/output, total peripheral resistance during PWA and pulse/heart rate (${2.47\le -\log }_{10}P\le 18.94$ and $0.05\le \left|r\right|\le 0.16$). In contrast, CV was considerably associated only with cardiac output (${-\log }_{10}P=2.88$, *r* = 0.06) and solely with LV ejection fraction (${-\log }_{10}P=3.21$, *r* = 0.06).

Comparing with QRSd, QTc interval was more strongly associated with only functional and hemodynamic phenotypes, such as cardiac index/output, total peripheral resistance during PWA and pulse/heart rate (${9.35\le -\log }_{10}P\le 56.35$ and $0.12\le \left|r\right|\le 0.28$), plus more hemodynamic phenotypes, including diastolic/systolic blood pressure, central systolic/pulse blood pressure during PWA and Arterial Stiffness Index ($2.34\le {-\log }_{10}P\le 9.91$ and $0.05\le \left|r\right|\le 0.13$). ${G}_{\rm{KrKs}}$ was associated with all QTc-associated phenotypes ($2.78\le {-\log }_{10}P\le 61.23$ and $0.05\le \left|r\right|\le 0.28$) and also with structural phenotypes, including LV end-diastolic, end-systolic and stroke volumes ($2.78\le {-\log }_{10}P\le 3.59,r=0.06$).

The biventricular myocardial mass was considerably associated with all phenotypes mentioned above (${-\log }_{10}P > 6.67$ and $\left|r\right|\ge 0.09$) where higher correlations were seen for structural phenotypes such as LV stroke, end-diastolic and end-systolic volumes (${-\log }_{10}P > 108$ and $\left|r\right|\ge 0.38$).

QRSd, CV and biventricular mass were weakly associated with mental health factors such as ‘Seen doctor (GP) for nerves, anxiety, tension or depression’ and neuroticism score ($2.35\le {-\log }_{10}P\le 3.92,\,0.05\le \left|r\right|\le 0.07$) (Supplementary Table 9). Other interesting associations with lifestyle phenotypes were identified, the details of which are in Supplementary Table 10. For example, using vitamin supplement was strongly associated with increased ${G}_{\rm{KrKs}}$ (${-\log }_{10}P=3.9$, *r* = 0.18).

### Association with clinical diagnoses

**Fig. 6:**
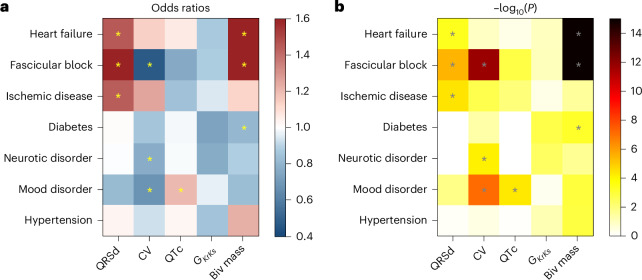
Association of QRSd, CV, QTc interval, baseline ‘${\mathbf{G}}_{\mathbf{KrKs}}$’ conductance and myocardial mass with common diseases. **a**, ORs for phenotypes as input risk factors for common diseases as the outcomes. Sex, age, BMI, age × BMI and sex × age were adjusted in the logistic regression analysis (*N* = 3,461). **b**, The corresponding *P* values (two-sided *t*-test) for ORs. *Result reaches Bonferroni threshold (${{{P}}}_{{\rm{Bonf}}}={7.1\times 10}^{-4}$ for ${\rm{\alpha }}$ = 0.05). Biv, biventricular. Source data

We investigated the associations of the multimodal and CDT-derived phenotypes with seven common diseases, categorized using the summary diagnoses for hospital inpatients. We trained a logistic regression model on QRSd, QTc, myocardial mass and CDT-derived CV and ‘${G}_{\rm{KrKs}}$’ conductance to predict disease occurrence, adjusted with demographics/anthropometrics factors, including sex, age, BMI, age × BMI and sex × age (Fig. 6). Supplementary Table 11 presents the odds ratios (ORs) derived from the regression coefficients (represented as $\rm{{value}}_{[95 \%\,{confidence\; interval}]}$) and the corresponding *P* values. The performances of the trained models are presented in Supplementary Table 12.

Greater QRSd and myocardial mass were associated with a higher risk of both HF and FB (QRSd: ORs = 1.45 and 1.92, $P < {4\times 10}^{-4}$ and mass: ORs = 1.78 and 1.82, $P < {3\times 10}^{-308}$). In contrast, smaller CVs were only associated with a high risk of FB (ORs = 0.48, $P={1.7\times 10}^{-12}$). IHD was more likely to develop with greater QRSd (ORs = 1.44, $P={3.6\times 10}^{-5}$), in agreement with the observed larger QRSd in the clinical IHD cohort, whereas diabetes was more likely to develop with smaller mass (ORs = 0.81, $P={6.3\times 10}^{-4}$). Reduced CV was the only CDT-derived predictor for both neurotic and mood disorders (ORs = 0.77 and 0.68, $P < {7.6\times 10}^{-5}$), whereas increased QTc was associated with high risk of mood disorder (ORs = 1.2, $P={5\times 10}^{-5}$). ${G}_{\rm{KrKs}}$ had non-significant prediction of risks of all diseases stated above.

## Discussion

This study makes three major contributions. First, we developed a fully automated EP CDT generation workflow, using multimodal data including both medical imaging and clinically measured ECGs. This workflow is a large-scale biophysically detailed CDT workflow, benefiting from the utility of statistical and artificial intelligence tools to perform GSA.

Second, we demonstrated the ability of CDT-derived phenotypes to unveil the underlying biological mechanisms and elucidate the variability in observables, such as imaging and ECG phenotypes, across populations by sex, age and BMI.

Third, our PheWAS showed stronger associations between CDT-derived phenotypes and cardiac and mental health traits, such as depression, than traditional imaging or ECG phenotypes. Moreover, a separate test on clinical outcomes shows that decreased CV is associated with higher risks of neurotic and mood disorders.

These findings highlight the value of generating CDT-derived phenotypes in identifying biomarkers that could help identify potential therapeutic targets and evaluate the therapeutic potential (or side effects) of existing/emerging medications.

CDTs have been rapidly advancing in recent decades, leveraging their capacity to guide, inform, monitor, diagnose and prognose therapies and surgical interventions in many current prospective clinical studies, paving the way for moving into industrial and clinical settings^1^. This shift requires a step change in the speed, robustness, validation and uncertainty quantification in both anatomical and functional model creation workflows.

Automated workflows for creating anatomical model creation workflows using machine learning have been developed for large-scale studies such as the UKBB^21,22^; however, they typically produce surfaces rather than high-quality volumetric meshes. Existing volumetric mesh creation workflows, such as for atria^23^, ventricles^24^ and whole hearts^6^, have been applied only to smaller datasets ($\le 100$) owing to high computational costs and manual steps required. Here we report a fully automated volumetric mesh generation workflow that can create personalized anatomical meshes at scale within clinical timescales (~5 min per CDT). The absolute difference of our reconstructed personalized biventricular meshes is consistent with previous studies^10^ (LVEDV: $-2.6\pm 11.3\; \;\rm{ml}$ and RVEDV: $-6.0\pm 9.4\; \;\rm{ml}$) and is attributed to the difference in volume integration performed in the apical region of the anatomy.

Functional model personalization at scale is more challenging, where most current modeling studies were developed using ‘average’ material property values from broader physiological studies with limited personalization^25,26^. Recent developments in high-fidelity EP CDT frameworks incorporated features such as His–Purkinje fascicles to replicate detailed QRS complex morphology, but only a subset of parameters underwent personalization yet still demanding substantial computational resources and time^24^. Alternative computationally efficient approaches based on machine learning and statistical methods were reported but often fall short in capturing all anatomical/functional details and struggle to generalize effectively^27^.

To move to population-level studies, it is of critical importance to balance biophysical fidelity, parameter inference and computational cost. In this work, instead of making CDTs to replicate recorded ECG morphologies, which are more likely to be afflicted by subject-specific noises, we constructed feature-specific CDTs to replicate the QRSd and QTc, which can be extracted from models robustly and computationally efficiently^28^. Similar to previous frameworks^24^, our framework incorporates parameters encapsulating knowledge derived from physiological and histological/anatomical experiments in the past. Our sensitivity analysis suggests that an optimal tradeoff between model fidelity and parameter identifiability is to restrict the EP personalization to one single parameter (CV and ${G}_{\rm{KrKs}}$), ensuring its computational efficiency (5 + 40 min per CDT).

Three model validations have been sought to assess the fidelity and validity of our CDTs (that is, which aspects of the real-world system are sufficiently recapitulated?). First, we compared how well our simulated 12-lead ECGs reproduce the recordings lead-wisely by conducting correlation tests on the 10 representative individuals. We found good 79% correlations when considering the top six ECGs in averaging all individuals, within clinical correlation thresholding^20^. We also found 87% correlations in vectorcardiogram (VCG) dipole magnitudes and a $15^\circ$ deviation in dipole orientations, similar to previous studies^29^, although we have standardized the ECG electrode locations, which have been identified as a key factor influencing QRS complex morphology but do not affect QRSd^26^. Second, our CDT-derived phenotypes were tested to differentiate individuals afflicted with FB or HF. Both conditions are known to be associated with slow conduction substrates and/or alternations in ionic channels, such as potassium. We found that the CVs for both patient groups are slower than the counterparts by 16.8% and 5.2% (Fig. 3), although we used a unified healthy conduction system in QRSd calibration. The ${G}_{\rm{KrKs}}$ is smaller in patients with HF, consistent with potassium channel downregulation in HF^30^. Third, we further validated our modeling approach by applying it to an independent clinical cohort of patients with IHD. We found consistent changes in CDT features with age, sex and BMI. An additional positive validation result is the fact that the separate association test on clinical outcomes identified that lower CV is associated with higher risks of FB (Fig. 6). Furthermore, in a verification test, we quantified the uncertainty of the inferred CVs and ${G}_{\rm{KrKs}}$, considering the uncertainty in assuming all other parameters (EP and ECG electrode positioning) as reference prior values, which further increases the credibility of our approach.

By building CDTs, we have been able to separate the impact of CV/${G}_{\rm{KrKs}}$ and heart anatomy on QRSd/QTc and quantitatively assess their relative changes across different populations, unveiling the underlying biological process. Consistent with previous studies^31^, we found that males have longer QRSd and larger myocardial mass than females, which can entirely be explained by the change in anatomy, with no discernible disparity in CVs, suggesting no significant biological differences. Accordingly, clinical guidelines that use QRSd as criteria, such as cardiac resynchronization therapy, can develop sex-specific thresholds of decision based on heart size differences and disregard the potential impact of CV variations. This result brings additional evidence to support the proposal of reducing the threshold for CRT in female individuals by 9–13 ms (ref. ^32^). Additionally, we observed a modestly longer QTc interval (3%) in females compared to males, alongside a considerable decrease in ${G}_{\rm{KrKs}}$ (−18%). This is consistent with previous findings of lower expression levels of certain proteins in females, such as hERG for ${I}_{\rm{Kr}}$ and MinK for ${I}_{\rm{Ks}}$, which are reduced by approximately 30%^33^. These results suggest a substantially smaller ionic channel density in females compared to males.

Longer QRSds were observed in overweight and obese compared to healthy groups, consistent with literature^34^, which are primarily attributed to the increased myocardial mass, a structural remodeling, shown to be casually associated with higher BMI^35^. Obesity may also lead to electrical remodeling, including conduction slowing and conduction heterogeneity, which are often observed in diseased clinical cohorts^36^. In contrast to earlier findings from clinical cohorts, we observed a concurrent small but substantial elevation in CVs corresponding to the increase in BMI. This suggests a potential adaptive response by the heart, possibly compensating for the effects of heightened myocardial mass on QRSd within obese groups. This mechanistic insight, initially identified in our study, warrants further investigations to delve deeper into this adaptive mechanism and its implications. We found that the increase of QTc in the overweight/obese group was due to reduced ${G}_{\rm{KrKs}}$, consistent with previous findings that alterations in ${I}_{\rm{Ks}}$ and ${I}_{\rm{Ks}}$ contribute to prolonged repolarization in obesity^37^.

We also observed longer QRSd in the elderly groups, which is mainly because of the decrease in CV. This decline in the CV may be driven by well-established age-related cellular and tissue-level remodeling, including impaired sodium channel function^38^ (potentially through loss-of-function genetic mutations such as *SCN5A* as identified in previous ECG age-delta genome-wide association studies^39^ and experimental studies^40^) and the development of myocardial fibrosis^21^. It may also be affected by gap junction decoupling such as connexin 43 downregulation, which is a known pathological remodeling in patients with ventricular hypertrophy and IHD^41^. We also observed aging associated with increased QTc, as observed previously^42^, but not with ${G}_{\rm{KrKs}}$, consistent with the absence of reports of changes in potassium channels with aging but worthy of future validation.

PheWAS is a data-driven method to generate new hypotheses based on the identification of correlations between exposure, such as genetic and environmental factors, and phenotypes, such as diseases and clinical outcomes. Unlike previous PheWASs focusing on either MRI-derived or genetic phenotypes^21,22,38^, our PheWAS included inferred tissue/molecule-level phenotypes—CV and ${G}_{\rm{KrKs}}$—uniquely estimated with our CDT approach, which can form a bridge between research findings on the genetic, molecular/cellular level to tissue and the whole organ level.

Despite a relatively smaller sample size (*N* = 3,461), we found that biventricular myocardial mass was highly associated with multiple structural phenotypes, such as LV stroke, end-diastolic and end-systolic volumes, as found in previously^22^. Interestingly, we found that, compared to the QRSd association with various MRI-derived phenotypes, CV was only positively associated with functional phenotypes: cardiac output and LV ejection fraction, which are clinically used to measure cardiac performance, particularly in the diagnosis of HF and post-myocardial infarction. Thus, CV may be used as a biomarker for disease evaluation and also as a potential therapeutic target for guiding the development of new drugs.

Unlike QRSd, QTc and ${G}_{\rm{KrKs}}$ were more strongly associated with many functional and hemodynamic phenotypes, whereas ${G}_{\rm{KrKs}}$ was also positively associated with structural phenotypes, such as LVEDV. This positive association of ${G}_{\rm{KrKs}}$ with heart size is consistent with the observed smaller ${G}_{\rm{KrKs}}$ in females who usually have smaller hearts compared to males.

We also found correlations of CV, QRSd and biventricular mass with mental health factors (>FDR threshold), whereas, in the separate association test on clinical outcomes, we found that only reduced CV was substantially associated with increased risks of neurotic and mood disorders. We also looked for links among CDT-derived, ECG and structural phenotypes with medications reported in the UKBB, but none, including mental health medications, had a significant association. Previous research established a bidirectional relationship between depression and cardiovascular diseases^43^. Increased evidence has linked psychological disorders with altered cardiac morphology and functions—for example, reduced LV mass, increased myocardial fibrosis^44^ which were also observed as changes in ECG metrics such as heart rate, QT interval, QRSd but not in all^45,46^. Other interesting correlations with environmental phenotypes, such as lifestyle and diet, have been identified as well (>FDR threshold)—for example, using vitamin supplement and increased ${G}_{\rm{KrKs}}$ (*r* = 0.18). Several plausible biological mechanisms between vitamins and cardiac ion channel function have been proposed, including antioxidant effects from vitamins C and E^47^ and gene expression regulation from vitamin D^48^. However, further mechanistic studies are needed to elucidate their relationships and to validate clinically.

Overall, the CDT-derived phenotypes exhibited greater sensitivity to functional alternations compared to existing structural and ECG phenotypes. This heightened sensitivity could be attributed to the fact that they are directly modulated by the physical properties of cardiac myocytes and their interconnections. Specifically, the changes in CV may more accurately reflect cardiac remodeling such as fibrotic alterations resulting from the decoupling of cell–cell connections and coupling of myocytes with fibroblasts^49^, and the changes in ${G}_{\rm{KrKs}}$ reflect the ionic remodeling, potentially leading to arrhythmogenesis^50^.

In conclusion, we provide a proof-of-concept cross-sectional study that demonstrates the potential for applying a CDT workflow to larger cohorts. This approach may yield deeper insights into the biological connections between changes in cardiac morphology and function with neurological disorders. Further validation can be pursued through longitudinal studies using resources such as the UKBB’s repeat imaging, and causal relationships can be established through large-scale genetic studies, such as the emerging area of heart–brain connection.

## Methods

### CDT creation

CDTs were created from the UKBB and an independent clinical validation dataset. The UKBB is an open-access resource accessed under application number 88878. Ethical approval was obtained from the Northwest Research Ethics Committee (REC reference: 11/ NW/0382), and written consent was obtained from all participants. The present study was performed on the first 4,326 participants with consent from the UKBB with adequate geometrical information^10^ and reported QRSd, QTc interval, sex, age, BMI/weight and height information. The details of the selection of population sample size and quality control were described previously^10,51^. The clinical validation cohort^52^ includes patients with IHD referred to Royal Brompton & Harefield NHS Hospital. These patients have undertaken cine cardiac magnetic imaging, 12-lead ECGs with reported demographics.

### Anatomical mesh generation

For the UKBB cohort, we used a nnU-net-based architecture^9^ for automatic segmentation of the LV and RV blood pools and LV myocardium, trained based on manual segmentations on short-axis heart images^51^. The end-diastolic (ED) phase was selected as the first phase of acquisition. Then, the contours and landmarks of LV and RV derived from the segmentations on the ED frame were extracted using an automatic contouring method. For the IHD cohort, CVI42 (Circle Cardiovascular Imaging, Inc.) was used for automatic segmentation and contour extraction, with manual clinical adjustment where necessary. Then, an atlas-based pipeline (previously validated)^10^ was used to construct personalized biventricular surface meshes. The RV epicardium was estimated by extending the RV endocardium points normal to the surfaces by 3 mm, consistent with experimental measurements^53,54^. The surface meshes were used to construct tetrahedral finite element meshes using Meshtool^55^ including regions of LV myocardium, RV myocardium, aortic, tricuspid, pulmonary and mitral valves. The biventricular myocardial mass was computed from the myocardial volume using a density of $1.05\;{\rm{g}\;{ml}^{-1}}$.

To enable automated computation for CDTs, a morphological coordinate system, known as universal ventricular coordinates (UVCs), was introduced for describing positions within ventricles based on the apical-basal ($Z$), transmural ($\rho$) (from endocardium to epicardium), rotational ($\varphi$) (anterior, anteroseptal, inferior, inferolateral and anterolateral) and chamber-wise (LV and RV) coordinates. Biventricular myocardial fiber structure was implemented using a rule-based approach with a transmural variation of angle $\alpha$ as from $60^{\circ}$ to −$60^{\circ}$ in longitudinal fiber directions and angle $\beta$ as from $-65^{\circ}$ to $25^{\circ}$ in transverse fiber directions from endocardium to epicardium. We also visually examined the generated fibers and UVCs for a subset of 50 individuals who were randomly sampled from the whole cohort.

### EP model framework

**Extended Data Fig. 9:**
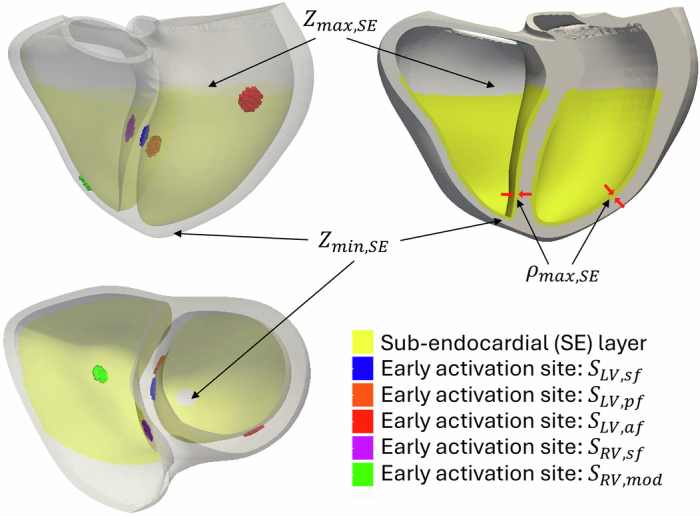
Fascicular-based model illustration replicating the realistic ventricular activation modulating by the His-Purkinje system. The SE layer (yellow) represents the fast conduction regions, where the fascicles are located, defined by apical-basal coordinate $Z$ and transmural coordinate $\rho$, bounded by physiological measurements from literature as shown in Supplementary Table 2. There are also five early activation sites represented by disks with a thickness of 5% of the ventricular wall (${\delta }_{z}$ and ${\delta }_{\rho }$ = 0.05) and having a fixed radius of $20\mu m$, representing ~25 cells. The disks are centred at root locations, defined by apical-basal coordinates $Z$ and rotational coordinates $\varphi$, which are activated at specific timings. Both coordinates and timings of the five sites are bounded by physiological measurements from literature as shown in Supplementary Table 2.

The EP simulations were performed using the Cardiac Arrhythmia Research Package (CARP)^56^. We used a reaction-eikonal model without diffusion to compute the sinus ventricular activation times and transmembrane potential transient over time^57^. The activation wavefront propagation in the myocardium $\Omega$ is described as:

1 $$\left\{\begin{array}{ll}\sqrt{\nabla {t}_{a}^{T}{\bf{V}}\nabla \,{t}_{a}}=1\;{{\rm{in}}\; \Omega }\\ {t}_{a}={t}_{0}\;{\rm{in}\; \Gamma }\end{array}\right.$$

where ${t}_{a}$ is the local activation time at any location in the myocardium; ${t}_{0}$ are the instants of initial activation at locations $\Gamma$; and the tensor field ${\bf{V}}$ encodes the spatially heterogeneous orthotropic squared CV. The ventricular myocardium was treated as transversely isotropic conductors. The ten Tusscher ionic model was used to simulate EP dynamics in the ventricular myocytes^16^. The ventricular depolarization during sinus rhythm is initiated by the His–Purkinje system (HPS). As direct measurement of the HPS in the cohort is not available, a fascicular-based model was used to represent the emergent physiological features of the HPS^58^. Overall, the EP framework of depolarization consists of 20 EP parameters with uncertainty as shown in Extended Data Fig. 9 and Supplementary Table 1.

The ventricular repolarization wave occurs after depolarization and is primarily determined by the dispersion of repolarization. This dispersion is driven by the activation sequence and heterogeneity in APD across the ventricles, resulting from spatial variations in ionic channel densities modulated by mRNA and protein expression.

The variation in ionic channel densities have been estimated in the latest four physiological-based human ventricular cell models: the ten Tusscher model^16^, the Grandi model^59^, the O’Hara model^60^ and the ToR-Ord model^61^. These models focus solely on transmural heterogeneity. From these models, we identified 14 ionic parameters that varied spatially across the heart (difference in endocardial and epicardial cells) as shown in Supplementary Table 4. The four cell models are parameterized based on different experimental datasets. This results in model-specific differences in the effect of specific ion channels on the APD. Specifically, the APD depends predominantly either on the slow delayed rectifier potassium current ${I}_{\rm{Ks}}$ in the ten Tusscher model or on rapid delayed rectifier potassium current ${I}_{\rm{Kr}}$ in the O’Hara model^13^. To address the inconsistent importance of these two currents, we introduced a common scaling parameter, ${G}_{\rm{KrKs}}$, for both the slow and rapid delayed rectifier potassium conductances. We performed a sensitivity analysis on the 13 cellular parameters, which included 12 ionic pathway conductances and the common scaling parameter ${G}_{\rm{KrKs}}$, to evaluate their impact on the APD—the key cellular characteristic affecting the QTc interval—using the ten Tusscher model. This analysis identified ${G}_{\rm{KrKs}}$ as the most critical cellular parameter for determining the APD, as demonstrated in Extended Data Fig. 3a.

To extend our findings from cellular to organ-level considerations, we also accounted for spatial heterogeneity across the ventricles. Thus, we introduced three additional parameters: the baseline ${G}_{\rm{KrKs}}$, the transmural gradient and the apex-to-base gradient.

The range for the baseline ${G}_{\rm{KrKs}}$ value was determined by estimating the values that yielded a physiological APD ranging from 250 ms to 450 ms, based on previous human measurements^16–19^. The ranges for the transmural and apex-to-base gradient parameters were assumed to be equal due to limited data on the apex-to-base gradient and were derived from reported transmural gradients from prior human cell models (Supplementary Table 4). In summary, the repolarization framework considered 13 parameters in the cell model and three in the whole heart model, including a total of 16 parameters.

Computing ECGs requires information on the position of the heart within the torso; however, this information was not available. The heart models were, therefore, registered to a heart enclosed in an existing torso model^28^ using the UVCs. In this torso model, the locations of electrodes used in measuring 12-lead ECGs were identified; the corresponding extracellular potentials were simulated; and the ECGs were computed. The QRSd was computed by finding the timepoints at which the spatial velocity exceeds 0.15 of the maximum spatial velocity in the reconstructed corresponding VCG from 12-lead ECGs, which fuse the information in all ECG traces^28^. The QTc intervals were computed by using the Q start time detected in the previous step and the T end time detected by finding the timepoint at which the dipole magnitude of the VCG drops below 0.05 after T peak. This QTc interval measurement algorithm was visually inspected for 500 simulated ECGs to ensure its accuracy and robustness.

The use of a representative torso introduced extra uncertainty regarding the relative locations of the 10 ECG electrodes within the real torsos. To quantify this uncertainty, we introduced another 30 parameters (Supplementary Table 2), which describe the variation of the Cartesian coordinates for the 10 electrodes, and assumed that each Cartesian coordinate can vary $\pm \,5\;\rm{cm}$ sufficiently to take account of all possible variations in electrode locations^62^.

In summary, the entire EP framework considered 65 parameters with uncertainty, including 20 tissue-level characteristics for depolarization (Supplementary Table 1), 16 parameters for repolarization (Supplementary Table 4) and 30 additional parameters for the electrode locations (Supplementary Table 2).

### GPE and GSA

To build personalized CDTs, we need to set 65 parameters. However, the clinical measurements needed to constrain these parameters are not available, and classical calibration techniques are prohibitive due to the massive computational costs required per case and the number of cases that we need to calibrate. We use GPEs as surrogate models to accelerate the evaluation of the effect of the input parameters on the model output of interest: QRSd and QTc interval. This allows us to (1) gain important mechanistic knowledge about the input–output interactions and (2), through a GSA, exclude the parameters that have little/no effects on the output, speeding up the personalization pipeline.

The training of GPEs was described previously^63^ by maximizing the model log-marginal likelihood using the GPErks emulation tool (http://github.com/stelong/GPErks). The GPE was defined as the sum of a deterministic mean function $h(x)$ and a Gaussian process $g(x)$. The mean function $h(x)$ is:

2 $$h\left(x\right)={\beta }_{0}+\mathop{\sum }\limits_{i=1}^{D}{\beta }_{i}$$

where ${\beta }_{i}$ are weights for input parameters $x=({x}_{1},\ldots ,{x}_{D})$.

The Gaussian process $g(x)$ is:

3 $$g\left(x\right) \sim {GP}\left(0,{{\rm{k}}}\left({{x}},{{{x}}}^{{\prime} }\right)\right)$$

We chose to use the squared exponential kernel as the kernel function ${\rm{k}}({{x}},{{x'})}$ for parameter dimensions $D$ less than 30 and the Matérn kernel to be the kernel function ${\rm{k}}({{x}},{{x'})}$ for parameter dimensions $D$ more than 50, considering the non-smoothness in the high-dimensional parameter space.

We evaluated the accuracy of GPEs using both coefficient of determination ${R}^{2}$ and independent standard error (ISE). The ${R}^{2}$ score is calculated as:

4 $${R}^{2}{\rm{:= }}1-\frac{{\sum }_{i=1}^{n}{\left({y}_{i}^{\rm{true}}-{y}_{i}^{\rm{mean}}\right)}^{2}}{{\sum }_{i=1}^{n}{\left({y}_{i}^{\rm{true}}-\bar{y}\right)}^{2}}$$

5 $$\bar{y}{\rm{:= }}\frac{1}{n}\mathop{\sum }\limits_{i=1}^{n}{y}_{i}^{\rm{true}}$$

where ${y}_{i}^{{true}}$ is the true outputs, $\bar{y}$ is the mean of true outputs and ${y}_{i}^{{mean}}$ is the predicted posterior mean of emulator outputs for $i=1,\ldots ,n$.

The ISE is calculated as:

6 $${ISE}:=\frac{100}{n}\bullet \mathop{\sum }\limits_{i=1}^{n}\left(\frac{\left|\,{y}_{i}^{{true}}-{y}_{i}^{{mean}}\right|}{\sqrt{{y}_{i}^{\mathrm{var}}}} < 2\right)$$

where ${y}_{i}^{\rm{true}}$ is the true outputs, and ${y}_{i}^{\rm{mean}}$ and ${y}_{i}^{\mathrm{var}}$ are the predicted posterior mean and variance of emulator outputs for $i=1,\ldots ,n$. The Boolean result inside the parentheses is encoded with either 0 (false) or 1 (true).

The ${R}^{2}$ evaluates the error between the predictions and the observations with close to 1 indicating a lower error. The ${\rm{ISE}}$ accounts for the distance between predictions to the true values and quantifies the GPE uncertainty. If $\rm{ISE}$ is close to 100%, it means that the true values are falling in the region of the predictions within 2 s.d.

We then performed a variance-based GSA^64^ on the trained GPEs to quantify the total effect for each input parameter (${S}_{T}$) on QRSd and QTc interval. The total effect consists of the first-order effect and the higher-order interactions, which are computed using the Saltelli method^64^ with the SALib Python library by taking *N* = 1,000 samples from the posterior distribution of trained GPEs. This allowed us to account for the effect of GPE uncertainty on the sensitivity indices. From the GSA, we ranked the total effects $({S}_{T})$ of the input parameters to identify the key parameters explaining the majority variation in the output QRSd and QTc interval.

To explore the heterogeneity in the cohort in terms of sex, age and BMI, we chose to perform individual-specific GSAs on a cohort of representative individuals including both healthy individuals and patients with cardiovascular diseases. To select the representative individuals, we generated 10 samples by Latin hypercube sampling on basic characteristics, including sex, age and BMI, and identified 10 healthy individuals (Supplementary Table 13) and 10 with pathology, including five with FB and five with HF, who have the closest basic characteristics to the generated samples (Supplementary Table 3).

First, we trained GPEs for each individual with the output as QRSd and performed a GSA using the trained GPEs to identify the key input parameters explaining the majority variation in the output QRSd. We trained GPEs and performed GSA first on the 20 tissue-level EP parameters to exclude parameters having small effects on the output QRSd. Then, we combined the key EP parameters with the 30 parameters related to ECG electrode locations to identify the key input parameters within the entire EP framework that account for most of the variation in QRSd. Additionally, we performed GSA on the complete parameter set to assess their effects in the same analysis for both the 10 healthy individuals and the 10 individuals with pathology.

Second, we investigated the parameters affecting QTc interval. Given that APD is the most important cellular characteristic determining T-wave, we first performed a GSA of the APD on the densities of the cell model ionic pathway (channels, pumps and exchangers), as shown in Supplementary Table 4. We then performed a GSA of the QTc interval on the parameters describing the value and spatial variation (transmural and apex-to-base variation) of the most important ionic pathway densities, identified from the cell-scale GSA and the 50 parameters included in QRSd GSA. The ${R}^{2}$ and $\rm{ISE}$ of trained GPEs on QRSd and QTc interval for the 10 healthy individuals and those with pathology are shown in Supplementary Tables 14 and 3, respectively.

**Extended Data Fig. 10:**
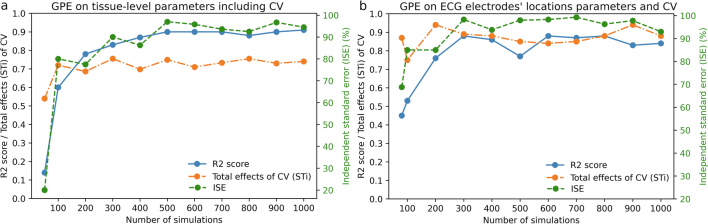
The impact of the number of simulations performed used for training the GPE on the resultant R2score and ISE along with the resultant total effects of CV. **a**. is for GPEs with the output as QRSd on the 30 tissue-level parameters. **b**. is for GPEs with the output as QRSd on 31 parameters including 30 ECG electrodes’ locations and CV. Source data

To train the GPEs for QRSd, we initially employed Latin hypercube sampling to obtain 300 samples for both the EP parameters and ECG electrode parameter sets. We then ran EP simulations for these samples to create the training dataset. The impact of the training dataset size on the GSA is illustrated in Extended Data Fig. 10. For the GPE trained with all parameters for QRSd, the sample size was 500 samples for 10 individuals with pathology, and it was increased to 1,500 samples for 10 healthy individuals for improving GPE accuracy. To train the GPEs for the QTc interval, we generated 530 samples.

### Personalized ECG metrics calibration

To facilitate the replication of the clinically measured QRSd and QTc interval in the UKBB across populations, a calibration workflow without any manual intervention is preferred. The GSA performed above enables us to identify the key input parameters responsible for the variation of QRSd and QTc interval and allows us to reduce the number of input parameters required for the calibration process. Here, we chose to vary only the most important parameter identified in the GSA for QRSd and QTc interval calibration, therefore allowing a computationally efficient bisection method to search for the subject-specific parameter (constrained by physiological measurements in literature) to match the corresponding QRSd and QTc interval.

To quantify the uncertainty due to varying only the most important parameter to ECG metrics—QRSd and QTc interval—we computed confidence intervals for the inferred parameter for the 10 individuals used in GPE training. First, we randomly sampled (*N* = 300) the other fixed parameters, assuming that they are from a normal distribution with a mean equal to the median of the physiological bounds and the upper and lower bounds set at mean plus or minus 3 s.d. (encompassing 99.72% of the data). Then, we performed simulations using the 300 samples combined with the subject-specific fitted parameter to estimate the potential range of the ECG metrics in each individual given the uncertainty in the uncalibrated 49 parameters. We then used the 5th and 95th percentiles of both ECG metrics as the targets to refit the key input parameter and then calculate their deviations from the original subject-specific fitted parameters, which represent the confidence interval of the inferred parameter, considering the variation of other fixed parameters. The median confidence intervals of the 10 individuals were then calculated and reported.

### QRS complex comparison between the simulated and recorded 12-lead ECGs in the UKBB

To assess the approximate errors of the simulated subject-specific 12-lead ECGs due to using a standard torso and fixed electrode locations in the personalized QRSd calibration, we compared the simulated 12-lead ECGs of 10 representative individuals across sex, age and BMI with their recorded ECGs in the UKBB. The simulated 12-lead ECGs were first filtered by the same filters as used in processed recorded ECGs in the UKBB. It includes a low-pass filter at 100 Hz, a high-pass filter at 50 Hz and a notch filter at 50 Hz. Then, each lead of simulated ECGs was temporally aligned to the corresponding recorded lead ECG by matching the timepoints that the maximum energy was achieved (${V}^{2}$) and was also scaled in amplitude to obtain the same maximum absolute values as in the recorded ECG. Each lead of the recorded ECG was also cropped to have the same length as the aligned and scaled simulated lead ECG. Finally, each pair of simulated and recorded lead ECGs was compared by computing the Pearson correlation coefficient ($r$). For each individual, the 12-lead ECGs were ranked by $r$, and the average $r$ was calculated by considering different numbers of ranked ECG leads.

Moreover, we compared the simulated and recorded VCGs, which are less dependent on the positioning of electrodes. We used the Kors transformation to derive the VCGs from both simulated and recorded ECGs, as in previous studies^28,29^. We first compared the magnitudes of simulated and recorded VCG dipoles by computing the Pearson correlation coefficient ($r$). Second, we compared the deviation $\theta$ in dipole orientation between the simulated and measured cardiac dipoles as:

7 $$\theta =\frac{180^\circ }{{t}_{\rm{total}}}\mathop{\sum }\limits_{t=0}^{{t}_{\rm{total}}}\frac{{\cos }^{-1}\left(\frac{{\widetilde{v}}_{t}^{\rm{simulated}}\,\bullet \,{\widetilde{v}}_{t}^{\rm{recorded}}}{\left|{\widetilde{v}}_{t}^{\,\rm{simulated}}\right|\left|{\widetilde{v}}_{t}^{\rm{recorded}}\right|}\right)}{\pi }$$

where at the time *t*, ${\widetilde{v}}_{t}^{\,\rm{simulated}}$ and ${\widetilde{v}}_{t}^{\rm{recorded}}$ are the simulated and recorded dipole vectors, and the ${t}_{{total}}$ is the total time of the aligned QRS complex.

### Statistical analysis

Statistical analysis was performed using the Python statsmodels library. The results are presented as mean ± s.d. unless specified. BMI was calculated from height and weight measures taken at the time of the MRI being taken. Codes for the UKBB fields are included in brackets. Age was computed using the year of birth (34), month of birth (52) and date of attending the assessment center (53) to get the actual age when imaging occurred. The Mann–Whitney *U*-test was used to compare two different groups, as the data were not normally distributed. In the PheWAS, we used a similar approach as in ref. ^22^. Before computing univariate cross-correlation, effects such as age, sex, weight and height were regressed out of the multimodal phenotypes, as they may confound with many phenotypes. The phenotypes from the UKBB were normalized, and then univariate cross Spearman correlation was applied between the de-confounded multimodal phenotypes and the UKBB phenotypes. The UKBB phenotypes were categorized into 15 groups, including PWA (128), LV size and function (133), abdominal composition (149), primary demographics (1001), early life (1002), self-reported medical conditions (1003), lifestyle diet (1004), physical measures (1006), education employment (1007), mental health (1018), summary diagnosis derived from summary diagnoses for hospital inpatient (41270), lifestyle alcohol (100051), physical activity (100054), smoking (100058) and medication. The self-reported medical condition and summary diagnosis were processed to have each column representing one disease code sorted in ascending order, before using in PheWAS. The medications group consists of 27 phenotypes that are identified by searching the keywords of ‘medications’ and ‘substances’ in the UKBB, as shown in Supplementary Table 15. We cleaned the data before performing PheWAS by discarding phenotypes with more than 90% missing data, and, if two highly correlated phenotypes had a correlation coefficient greater than 0.9999, only one phenotype was kept.

### Reporting summary

Further information on research design is available in the Nature Portfolio Reporting Summary linked to this article.

## Supplementary information

Reporting Summary

Supplementary Tables 1–15 Supplementary Table 1 Twenty tissue-level EP characteristics with uncertainty with bounds based on literature. Note that the myocardial CV transverse to fiber direction can also vary but is assumed to be proportional to the CV along the fiber in the table with the anisotropy ratio as 0.42. Supplementary Table 2 Thirty ECG electrode position characteristics with uncertainty. Supplementary Table 3 Basic characteristics of 10 pathological cases sampled from the cohort, including five with FB and another five with HF, following with *R*^*2*^ *score* and ISE for GPEs trained on QRSd in those individuals. Code for sex: 0 means female, and 1 means male. Supplementary Table 4 The fold range changes of different ionic parameters between epicardium and endocardium based on experimental measurements conducted in four state-of-the-art ventricular ionic current models. Supplementary Table 5 Basic characteristics of participants from the UKBB. *N* = 3,461. The BMI was calculated from height and weight measures taken at imaging. Age was computed using the year of birth (34), month of birth (52) and date of attending assessment centre (53) to get the actual age when imaging. Disease codes are derived from summary diagnoses in the UKBB (41,270) as shown in Supplementary Table 2. The percentages of specific individuals in the whole cohort are shown in brackets. Supplementary Table 6 Comparison between the simulated and recorded VCGs in QRS complex for the 10 representative individuals. The first column shows the correlation of magnitude of simulated and recorded dipoles, and the second column shows the deviation *θ* in dipole orientation between the simulated and measured cardiac dipoles in degree. Supplementary Table 7 Common diseases categorized from summary diagnoses in the UKBB (41,270). Supplementary Table 8 Basic characteristics of the clinical cohort of patients with IHD. *N* = 359. Supplementary Table 9 The PheWAS associations to mental health factors. Columns 2 and 3 denote the *P* value (two-sided *t*-test) and Spearman’s correlation coefficient *r*. Supplementary Table 10 Other PheWAS associations for QRSd, CV, QTc and *G*_KrKs_. Columns 2 and 3 denote the *P* value (two-sided *t*-test) and Spearman’s correlation coefficient *r*. Supplementary Table 11 ORs (95% confidence interval) for phenotypes as a risk factor for a common disease as the outcome. The logistic regression analysis is adjusted for sex, age, BMI, age × BMI and sex × age as additional independent variables. Supplementary Table 12 Assessing the performances of the trained logistic regression models using the area under the receiving operating characteristic curve (AUC), accuracy, F1 score, sensitivity and specificity. The results are assessed on the 20% testing sets from the whole data. Supplementary Table 13 Basic characteristics of 10 representative healthy individuals sampled from the UKBB. Code for sex: 0 means female, and 1 means male. Supplementary Table 14 R^2^ score and ISE for GPEs trained on QRSd in 10 individuals, first on tissue-level parameters, second on ECG electrodes’ locations and CV. The third is for whole parameters on QRSd, and the last is for all parameters on QTc interval. Supplementary Table 15 The phenotypes in the medications group, identified by searching the keywords of ‘medications’ and ‘substances’ in the UKBB.

## Source data

Source Data Fig. 2 Statistical source data.

Source Data Fig. 5 Statistical source data.

Source Data Fig. 6 Statistical source data.

Source Data Extended Data Fig. 1 Statistical source data.

Source Data Extended Data Fig. 2 Statistical source data.

Source Data Extended Data Fig. 3 Statistical source data.

Source Data Extended Data Fig. 4 Statistical source data.

Source Data Extended Data Fig. 6 Statistical source data.

Source Data Extended Data Fig. 7 Statistical source data.

Source Data Extended Data Fig. 8a Statistical source data.

Source Data Extended Data Fig. 8b Statistical source data.

Source Data Extended Data Fig. 10 Statistical source data.

## Data Availability

The imaging data and non-imaging participant phenotypes and clinical outcomes are available from the UK Biobank via a standard application procedure at http://www.ukbiobank.ac.uk/register-apply. The CDT-derived phenotypes and the fitted ECGs for 10 healthy representative cases are returned to the UK Biobank (upload identifiers 6487 and 6537). The data for the ischemic heart disease cohort are patient data, and consent is not available to make this dataset publicly available. It can be accessed through reasonable request for an institutional data-sharing agreement.

## Code availability

The anatomical model generation pipeline is available at https://www.github.com/cdttk/biv-volumetric-meshing, which includes the use of open-source software: openCARP, available at https://opencarp.org/. The code of functional twininng pipeline, Gaussian process emulator training and global sensitivity analysis used in this study is available at https://github.com/cdttk/EPfitting_GPE. The functional twinning pipelines presented in this study were performed using the reaction-eikonal model, which is part of a proprietary version of the Cardiac Arrhythmia Research Package (CARP)^56^ and requires a license. However, this can also be performed using a monodomain model by openCARP, which is fully open source, albeit computationally more expensive.
