# Supplementary material for: Developing cardiac digital twin populations powered by machine learning provides electrophysiological insights in conduction and repolarization
Source: Nat Cardiovasc Res. 2025 May 16;4(5):624–36. doi: 10.1038/s44161-025-00650-0 (PMC12084159; doi:10.1038/s44161-025-00650-0)
Supplement: Supplementary file 1 — Reporting Summary [file 44161_2025_650_MOESM1_ESM.pdf]

nature portfolio

Corresponding author(s): Shuang Qian

Last updated by author(s): Feb 11, 2025

Reporting Summary

Nature Portfolio wishes to improve the reproducibility of the work that we publish. This form provides structure for consistency and transparency in reporting. For further information on Nature Portfolio policies, see our Editorial Policies and the Editorial Policy Checklist.

Statistics

For all statistical analyses, confirm that the following items are present in the figure legend, table legend, main text, or Methods section.

n/a	Confirmed	
☐	☒	The exact sample size (n) for each experimental group/condition, given as a discrete number and unit of measurement
☐	☒	A statement on whether measurements were taken from distinct samples or whether the same sample was measured repeatedly
☐	☒	The statistical test(s) used AND whether they are one- or two-sided<br>*Only common tests should be described solely by name; describe more complex techniques in the Methods section.*
☐	☒	A description of all covariates tested
☐	☒	A description of any assumptions or corrections, such as tests of normality and adjustment for multiple comparisons
☐	☒	A full description of the statistical parameters including central tendency (e.g. means) or other basic estimates (e.g. regression coefficient) AND variation (e.g. standard deviation) or associated estimates of uncertainty (e.g. confidence intervals)
☐	☒	For null hypothesis testing, the test statistic (e.g. F, t, r) with confidence intervals, effect sizes, degrees of freedom and P value noted<br>*Give P values as exact values whenever suitable.*
☒	☐	For Bayesian analysis, information on the choice of priors and Markov chain Monte Carlo settings
☒	☐	For hierarchical and complex designs, identification of the appropriate level for tests and full reporting of outcomes
☐	☒	Estimates of effect sizes (e.g. Cohen's d, Pearson's r), indicating how they were calculated

Our web collection on statistics for biologists contains articles on many of the points above.

Software and code

Policy information about availability of computer code

Data collection	No software was used for data collection.
Data analysis	The model generation pipeline will be made publicly available after publication, which includes the usage of open-sourced software: OpenCarp, available at https://opencarp.org/ and Meshtool, available at https://bitbucket.org/aneic/meshtool/src/master/. The code of functional twining pipeline, Gaussian Process emulator training and global sensitivity analysis used in this study is available at: https://github.com/cdttk/EPfitting_GPE.

For manuscripts utilizing custom algorithms or software that are central to the research but not yet described in published literature, software must be made available to editors and reviewers. We strongly encourage code deposition in a community repository (e.g. GitHub). See the Nature Portfolio guidelines for submitting code & software for further information.

Data

Policy information about availability of data

All manuscripts must include a data availability statement. This statement should provide the following information, where applicable:

- Accession codes, unique identifiers, or web links for publicly available datasets
- A description of any restrictions on data availability
- For clinical datasets or third party data, please ensure that the statement adheres to our policy

The imaging data and non-imaging participant phenotypes and clinical outcomes are available from UK Biobank via a standard application procedure at http://

www.ukbiobank.ac.uk/register-apply. The data for the ischemic heart disease cohort is patient data and consent is not available to make this dataset publicly available. It can be accessed through reasonable request for an institutional data sharing agreements.

Research involving human participants, their data, or biological material

Policy information about studies with human participants or human data. See also policy information about sex, gender (identity/presentation), and sexual orientation and race, ethnicity and racism.

Reporting on sex and gender	Sex-based analysis is performed in this study. The biological sex and self-reported gender were compared for the participants in this cohort.
Reporting on race, ethnicity, or other socially relevant groupings	Ethnicity for the participants were reported in the UK biobank. In this study, this information was not used for classification.
Population characteristics	Data was collected from two sources. First is from UK biobank which is a general population-based cohort in the United Kingdom in middle to old age. Supplementary Table 5 shows the population characteristics. Second is a clinical cohort of patients with ischemic heart disease (IHD) referred to Royal Brompton & Harefield NHS hospital. Supplementary Table 8 shows the population characteristics.
Recruitment	We used the 4,326 first participants with consent from the UK biobank that had adequate geometrical information along with reported QRSd, QTc, sex, age, BMI/weight and height information. The other clinical validation cohort includes 359 patients with ischemic heart disease (IHD) referred to Royal Brompton & Harefield NHS hospital.
Ethics oversight	UK Biobank has approval from the North West Research Ethics Committee (REC reference: 11/NW/0382). The registry of the IHD cohort complied with the Declaration of Helsinki, and the National Research Ethics Service approved the protocol.

Note that full information on the approval of the study protocol must also be provided in the manuscript.

Field-specific reporting

Please select the one below that is the best fit for your research. If you are not sure, read the appropriate sections before making your selection.

☒ Life sciences ☐ Behavioural & social sciences ☐ Ecological, evolutionary & environmental sciences

For a reference copy of the document with all sections, see nature.com/documents/nr-reporting-summary-flat.pdf

Life sciences study design

All studies must disclose on these points even when the disclosure is negative.

Sample size	No sample size calculation was performed. Due to the limited computational power available by the time the study conducted, we used the 4326 first participants with consent from the UK biobank that had adequate geometrical information and reported QRS duration, sex, age, BMI/weight and height information.
Data exclusions	We used 3461 subjects to perform the data analysis studies. 868 Subjects were excluded that did not had adequate geometrical information (allowing to go through the anatomical and functional personalization workflows) or did not have reported QRS duration, QTc interval, sex, age, BMI/weight and height information. In addition, we have used 359 patients who have available meshes constructed and adequate ECG, demongraphics information.
Replication	For the anatomical and functional personalization code, we carefully checked the code and perform model validation as in Results section. For the statistical findings in different sex, BMI and age groups, we compared our findings with pre-clinical and clinical studies that conducted on various different datasets. For the statistical findings in the exploratory PheWAS, we found similar results as in the previous study on imaging phenotypes from the UK biobank (doi: 10.1038/s41591-020-1009-y). Additionally, we selected significant correlations with mental-health phenotypes and followed on with a logistic regression analysis on related clinical outcomes that reveal significant and stronger associations of conduction velocity, surpassing the known phenotypes QRS duration, QTc interval and myocardial mass.
Randomization	UK Biobank is an observational prospective epidemiological study. The image analysis and statistical analysis in our study used the first 4326 subjects with CMR images and that fulfill the criteria described above. There was no process of randomisation that came into the analysis (this is not a controlled randomised study).
Blinding	This study is a retrospective study, rather than a randomized controlled trials (RCTs), therefore there was no blinding involved.

Reporting for specific materials, systems and methods

We require information from authors about some types of materials, experimental systems and methods used in many studies. Here, indicate whether each material, system or method listed is relevant to your study. If you are not sure if a list item applies to your research, read the appropriate section before selecting a response.

Materials & experimental systems

n/a	Involved in the study
☒	☐ Antibodies
☒	☐ Eukaryotic cell lines
☒	☐ Palaeontology and archaeology
☒	☐ Animals and other organisms
☒	☐ Clinical data
☒	☐ Dual use research of concern
☒	☐ Plants

Methods

n/a	Involved in the study
☒	☐ ChIP-seq
☒	☐ Flow cytometry
☒	☐ MRI-based neuroimaging

Plants

Seed stocks

Report on the source of all seed stocks or other plant material used. If applicable, state the seed stock centre and catalogue number. If plant specimens were collected from the field, describe the collection location, date and sampling procedures.

Novel plant genotypes

Describe the methods by which all novel plant genotypes were produced. This includes those generated by transgenic approaches, gene editing, chemical/radiation-based mutagenesis and hybridization. For transgenic lines, describe the transformation method, the number of independent lines analyzed and the generation upon which experiments were performed. For gene-edited lines, describe the editor used, the endogenous sequence targeted for editing, the targeting guide RNA sequence (if applicable) and how the editor was applied.

Authentication

Describe any authentication procedures for each seed stock used or novel genotype generated. Describe any experiments used to assess the effect of a mutation and, where applicable, how potential secondary effects (e.g. second site T-DNA insertions, mosiacism, off-target gene editing) were examined.
